# Progress and prospectus in genetics and genomics of *Phytophthora* root and stem rot resistance in soybean (*Glycine max* L.)

**DOI:** 10.3389/fgene.2022.939182

**Published:** 2022-11-14

**Authors:** Subhash Chandra, Mukesh Choudhary, Pravin K. Bagaria, Vennampally Nataraj, Giriraj Kumawat, Jeet Ram Choudhary, Humira Sonah, Sanjay Gupta, Shabir Hussain Wani, Milind B. Ratnaparkhe

**Affiliations:** ^1^ ICAR-Indian Institute of Soybean Research, Indore, India; ^2^ ICAR-Indian Institute of Maize Research, Ludhiana, India; ^3^ Department of Plant Pathology, Punjab Agricultural University, Ludhiana, India; ^4^ ICAR-Indian Agricultural Research Institute, New Delhi, India; ^5^ National Agri-Food Biotechnology Institute, Mohali, India; ^6^ Mountain Research Centre for Field Crops, Sher-e-Kashmir University of Agricultural Sciences and Technology, Srinagar, Jammu and Kashmir, India

**Keywords:** soybean, *Phytophthora*, disease resistance, gene stacking, sustainable management, genomic approaches

## Abstract

Soybean is one of the largest sources of protein and oil in the world and is also considered a “super crop” due to several industrial advantages. However, enhanced acreage and adoption of monoculture practices rendered the crop vulnerable to several diseases. Phytophthora root and stem rot (PRSR) caused by *Phytophthora sojae* is one of the most prevalent diseases adversely affecting soybean production globally. Deployment of genetic resistance is the most sustainable approach for avoiding yield losses due to this disease. PRSR resistance is complex in nature and difficult to address by conventional breeding alone. Genetic mapping through a cost-effective sequencing platform facilitates identification of candidate genes and associated molecular markers for genetic improvement against PRSR. Furthermore, with the help of novel genomic approaches, identification and functional characterization of *Rps* (resistance to *Phytophthora sojae*) have also progressed in the recent past, and more than 30 *Rps* genes imparting complete resistance to different PRSR pathotypes have been reported. In addition, many genomic regions imparting partial resistance have also been identified. Furthermore, the adoption of emerging approaches like genome editing, genomic-assisted breeding, and genomic selection can assist in the functional characterization of novel genes and their rapid introgression for PRSR resistance. Hence, in the near future, soybean growers will likely witness an increase in production by adopting PRSR-resistant cultivars. This review highlights the progress made in deciphering the genetic architecture of PRSR resistance, genomic advances, and future perspectives for the deployment of PRSR resistance in soybean for the sustainable management of PRSR disease.

## Introduction

Soybean (*Glycine max* (L.) Merrill) is an important legume crop that fulfills the substantial demand for food and feed globally. It is high in protein and oil content and also serves as a source of nutraceuticals such as bioflavonoids, lecithins, phytosterols, saponins, and tocopherols. Its oil is mainly used for domestic purposes; however, recent trends move toward the use of soybean oil as biodiesel to decrease reliance on fossil fuels (Mofijur et al., 2013). Approximately 70% of soybean’s economic value is for its meal, of which 97% is consumed as livestock and poultry feed (Raghuvanshi and Bisht 2010). The demand for soybean in the international market is increasing due to interest in functional food and the use of various soybean seed constituents and by-products in a wide array of specific industrial products (Kumawat et al., 2016). Globally, soybean is grown in an area of 122.6 million hectares (mha) with an annual average production of 336.6 million tons (mt) (USDA, 2020). The leading producers of soybean are Brazil, the United States, China, Argentina, and India (USDA, 2020). Like other food crops, soybean production is being challenged by various forms of abiotic and biotic stresses. The remarkable growth in the number of major diseases and their area has been observed in the past 50 years; subsequently, it negatively affects soybean production worldwide. The impact of diseases on soybean may be cited to the fact that the average annual economic loss due to soybean diseases in the US reached nearly $4.55 billion based on an investigation from 1996 to 2016 (Bandara et al., 2020). This increase in the number and spread of diseases can be attributed to enhanced acreage in new un-adapted regions and monoculture practices resulting in high pathogen density. Various factors governing the disease severity and economic losses include the pathogen type, plant tissue under attack, affected number of plants, the severity of an attack, pathogen-favoring environment, host plant vulnerability, plant stress level, and crop development stages (Hartman and Hill, 2010).

Among the various soybean diseases, Phytophthora root and stem rot (PRSR), caused by the soil-borne pathogen *Phytophthora sojae* (Kaufmann and Gerdemann) (oomycete pathogen), is the second most important economic disease after soybean cyst nematodes in the world. Earlier, *P. sojae* was part of the *Phytophthora megasperma* species complex which causes rot diseases in plants (Lin et al., 2021). *Phytophthora sansomeana* is identified as another causal agent for root rot in soybean, and partial resistance to *P. sansomeana* in soybean has been observed (Lin et al., 2021). Like *P. sojae, P. sansomeana* is also part of the *P. megasperma* complex. However, stem and root rot caused by *P. sansomeana* is not included in this review to keep the article length in check. PRSR drastically limits the yields of soybean globally as losses caused by it range between 10 and 40% or complete yield loss in some scenarios (Xiao et al., 2011; Zhang et al., 2013a). In the last few decades, *Phytophthora* root and stem rot resistance has been characterized by many researchers (Ryley et al., 1998; Dorrance et al., 2003; Grau et al., 2004; Sugimoto et al., 2006; Dorrance et al., 2008). In the United States, a loss of nearly 20.5 million tons was reported from 1996 to 2014, with an average annual loss of over 1.1 million tons due to this pathogen (Allen et al., 2017). *P. sojae* was first reported in Indiana state of the United States in 1948 (Kaufmann, 1957). Later, it spread to the major soybean growing areas of the United States, particularly in the pathogen-favoring environment of the Northern United States (Dorrance and Schmitthenner 2000). In addition to the United States, PRSR has been reported in other soybean-producing continents, namely, Asia, Africa, Australia, and Europe. The occurrence and development of PRSR are facilitated by poorly drained clay soils, low temperatures, and high rains (Kaufmann, 1957; Han et al., 2008). PRSR is generally characterized by the damping-off of seedlings and rotting of roots in adult plants (Tyler, 2007), and affected plants exhibit red–brown water-soaked lesions, wilting, and chlorosis, which in the case of extreme severity leads to mortality (Schmitthenner 1985; Dorrance et al., 2003).

*P. sojae* has abundant pathogenic diversity, and complete and partial resistance reactions have been reported for this pathogen. PRSR is being managed by cultivars with one or two dominant resistance genes for *Phytophthora sojae* named “*Rps”* (Jang and Lee 2020). However, the *Rps* genes are race-specific and useful as introgression of such genes is easy; but partial resistance has its own advantage for long-term protection. The first resistance gene against soybean *P. sojae* (named *Rps1a*) was identified in the 1950s (Bernard et al., 1957). Later, with the advent of sequencing technology and development of abundant simple sequence repeat (SSR) markers (Song et al., 2010), molecular linkage mapping gained pace, and nearly 30 *Rps* genes have been identified to date (Lin et al., 2013; Sun et al., 2014a; Ping et al., 2016; Niu et al., 2017; Zhong et al., 2019; Zhong et al., 2019; Jiang et al., 2020). In addition to SSR markers, a large number of single nucleotide polymorphisms (SNPs) and insertion/deletion markers for fine genetic mapping and molecular breeding have also been studied in different mapping populations (Li et al., 2016a; Li et al., 2017a). Functional characterization of identified genes has also gained progress in the recent past (Fan et al., 2015; Fan et al., 2017; Jang and Lee, 2020; Zhou et al., 2022). Few transcriptomic studies also uncovered molecular pathways in response to *P. sojae* infection (Guo et al., 2011; Lin et al., 2014). Newly identified genes for PRSR resistance serve as a good source for modern breeding programs to improve the resistance of cultivars to PRSR disease. Furthermore, the identified quantitative disease resistance loci (QDRL) can be employed in gene stock mining for the identification of novel alleles. This review aims to provide the current progress and future perspectives on genetics and genomics-assisted studies of *P. sojae R*-genes/QDRL and their utilization in soybean improvement.

## Disease management, pathogenic diversity, and potential genetic resources

PRSR is a serious concern today as it causes a significant yield loss in soybean production. Current PRSR control strategies include applications of various fungicides (Anderson and Buzzell, 1992), improving soil drainage systems (Schmitthenner, 1985), tillage systems (Workneh et al., 1998), application of calcium-containing fertilizers (Sugimoto et al., 2010), and the use of resistant varieties (Schmitthenner, 1999; Dorrance et al., 2003). Germplasm screening-based identification of resistant genotypes and development of PRSR-resistant soybean cultivars is the most effective and sustainable approach for minimizing yield losses (Burnham et al., 2003).

Currently, the management of PRSR is largely dependent on resistant cultivars, having one or more resistance genes. For understanding, pathogen race refers to a pathogen’s ability to cause disease in its host (Anderson et al., 2010); in other words, the pathogen race attacks certain resistance genes (Dorrance et al., 2016), and this kind of resistance is accompanied by several mechanisms including effector-triggered immunity (ETI), where R gene products in the host is recognized (directly/indirectly) by specific pathogen effectors termed avirulence (Avr) proteins (Li et al., 2021). Till now, nine *Avr* genes of *P. sojae* have been cloned (Yang et al., 2019). Soybean R genes whose products recognize *P. sojae* Avr effectors and trigger *Phytophthora* resistance are known as *Rps* (resistance to *P. sojae*) genes (Tyler and Gijzen, 2014). For understanding, avirulence 1c (*Avr1c*) gene in *P. sojae* confers the resistance by *Rps 1c* gene in soybean populations; the K105 amino-acid residue in *Avr* gene is the main determinant of the avirulence of *Avr1c* that interacts with *Rps* gene (Yang et al., 2019). So *Rps* genes have the potential to combat PRSR, but they are race-specific; therefore, they would be operational against limited *P. sojae* isolates, and each *Rps* gene often remains effective for about 8–15 years, which leads to the emergence of new isolates after a certain period (Schmitthenner 1985; Dorrance et al., 2003).

The diversity of the *P. sojae* population has been investigated in the United States and Canada since the 1960s, and most of the *P. sojae* isolates were determined based on studies with 15 host-differentials (Dorrance et al., 2004). During the 1980s, *Rps* genes *1a*, *1d*, and *1k* have been widely exploited to combat PRSR losses; however, the emergence of new isolates and enhancement in virulence lead to the evolution of more than 55 races reported against eight soybean differentials (*Rps1a*, *1b*, *1c*, *1d*, *1k*, *2*, *3a*, *6*, and *7* genes) (Abney et al., 1997; Grau et al., 2004). A total of 213 virulent pathotypes were identified from 873 isolates of the North Central United States (Dorrance et al., 2016).

In China, after the identification of *P. sojae* in the Heilongjiang region in 1991 (Su and Shen 1993), the incidence of the pathogen was reported mainly in the Inner Mongolia Autonomous Region, Xinjiang Uygur Autonomous Region, and Fujian Province till 2015 (Wen and Chen 2002; Chen et al., 2004; Liu et al., 2006; Xiao et al., 2011). During 2005–2007 in Heilongjiang Province, a total of 96 isolates were collected and investigated, which revealed that four out of the eight races had new pathotypes (Zhang et al., 2010).

PRSR was first reported in Hokkaido, Japan, in 1977 (Tsuchiya, 1990). Tsuchiya (1990) found the genetic differences between American and Japanese isolates during the investigation of 49 Japanese isolates and 55 known American *P. sojae* races. Sugimoto et al. (2006) collected 51 isolates from Hyogo in Japan and identified four new races. More than 100 *P. sojae* isolates were reported from 14 different regions for 14 *Rps* genes including *Rps1a*, *1b*, *1c*, *1d, 1k*, *3b*, *7*, and *8*; among them, *Rps1d* and *1k* were determined as the most promising resistance genes (Moriwaki 2010). Similarly, in Brazil, *P. sojae* was found to be different from other regions; 17 pathotypes were determined based on 37 Brazilian isolates, which were genetically different from the previously reported ones (Costamilan et al., 2013). Subsequently, *Rps1a*, *1c*, and *1k* were highly utilized in Brazilian soybean breeding programs, whereas *Rps1a* and *1c* were not effective in the United States (Costamilan et al., 2013). In South Korea, PRSR was first reported 2 decades ago (Jee et al., 1998). Kang et al. (2019) reported genetic differences among the pathotypes of Korean *P. sojae* isolates. Thus, based on these facts, it is a prerequisite to collect *P. sojae* isolates from several regions/fields and to assess them with differential varieties.

A number of genetic sources for *P. sojae* resistance have been identified and utilized to map *Rps* genes and to develop resistant cultivars through different breeding strategies (Table 1). Similarly, numerous genetic resources for incomplete or partial resistance for *P. sojae* have been utilized for genetic studies in the form of breeding lines and introgression (Table 2). Dorrance and Schmitthenner (2000) evaluated over 1,000 accessions from USDA germplasm accessions and found 162 accessions to be resistant to three races (7, 17, and 25). In addition to this, they also reported partial resistance in 55.5% of the 887 accessions for *P. sojae*. Kang et al. (2019) evaluated the *Rps* resistance against four isolates in 20 popular varieties of South Korea, while Daewon was identified as a resistant cultivar.

**TABLE 1:** Details of *P. sojae-*resistant genes (*Rps*), their source, chromosomal positions, and associated markers.

S. No.	Name of *Rps* gene	Chr no. (LG)	Source	^a^Position 1 (Mbp)	^a^Position 2 (Mbp)	Flanking marker 1	Flanking marker 2	References
1	*Rps1a*	3 (N)	L88-8470, Mukden, and Harlon	3.2	3.9	Satt159 (BARCSOYSSR_03_0180)	Satt009 (BARCSOYSSR_03_0226)	Bernard et al. (1957), Weng et al. (2001)
2	*Rps1b*	3 (N)	L77-1863	3.4	5.7	Satt152 (BARCSOYSSR_03_0192)	Satt530	Mueller et al. (1978), Demirbas et al. (2001)
3	*Rps1c*	3 (N)	L75-3735	3.4	9.2	Satt152 (BARCSOYSSR_03_0192)	Satt584 (BARCSOYSSR_03_0442)	Mueller et al. (1978), Demirbas et al. (2001)
4	*Rps1d*	3 (N)	L93-3312 and PI 103091	3.4	3.5	Satt152 (BARCSOYSSR_03_0192)	Sat_186 (BARCSOYSSR_03_0204)	Buzzell and Anderson (1992), Sugimoto et al. (2008)
5	*Rps1k*	3 (N)	L77-1794, Williams82, and E00003	-	-	CG1 (AFLP)	-	Bernard and Cremeens (1981), Kasuga et al. (1997), Gao and Bhattacharyya (2008)
6	*Rps2*	16 (J)	L76-1988, P I398440, and P I398694	1.64	34.03	Satt287 (BARCSOYSSR_16_0090)	Satt547 (BARCSOYSSR_16_1165)	Kilen et al. (1974), Demirbas et al. (2001), Gordon et al. (2007)
7	*Rps3a* *^b^*	13 (F)	L83-570, P I3 99036, PI408097, and PI424354	23.68	37.6	Satt374 (Sat_309)	Satt144	Mueller et al. (1978), Demirbas et al. (2001), Gordon et al. (2007)
8	*Rps3b*	13 (F)	L91-8347	-	-	-	-	Ploper et al. (1985), Demirbas et al. (2001)
9	*Rps3c*	13 (F)	L92-7857	-	-	-	-	Sugimoto et al. (2012), Demirbas et al. (2001)
10	*Rps4*	18 (G)	L85-2,352, and PI399036	53.8	56.3	Satt191 (BARCSOYSSR_18_1750)	Sat_064 (BARCSOYSSR_18_1858)	Athow et al. (1980), Demirbas et al. (2001), Sandhu et al. (2004), Gordon et al. (2007)
11	*Rps5*	18 (G)	L85-3059 and PI399036	-	53.9	-	Satt472 (BARCSOYSSR_18_1708)	Buzzell and Anderson, 1981; Sahoo et al., 2017
12	*Rps6*	18 (G)	L89-1,581, PI399079, and PI399036	54.5	-	Satt191 (BARCSOYSSR_18_1750)	Sat_372	Athow and Laviolette (1982), Gordon et al. (2007)
13	*Rps7*	3 (N)	L93-3258, OX281, and PI408097	3.9	18.4	Satt009 (BARCSOYSSR_03_0226)	Satt125 (BARCSOYSSR_03_0564)	Anderson and Buzzel (1992), Weng et al. (2001), Gordon et al. (2007)
14	*Rps8*	13 (F)	PI 399073	24.3	28.9	Satt425 (BARCSOYSSR_13_0784)	Satt114 (BARCSOYSSR_13_1055)	Gordon et al. (2004), Gordon et al. (2006), Sandhu et al. (2005)
15	*Rps9*	3 (N)	Ludou 4 and Cangdou 5	2.94	3.15	Satt631 (BARCSOYSSR_03_0162)	Sat_186 (BARCSOYSSR_03_0204)	Wu et al. (2011a)
16	*Rps10*	17 (D2)	Wandou 15	30.8	31.1	Sattwd15-24	Sattwd15-47	Zhang et al. (2013a)
17	*Rps11*	7 (M)	PI 594527	5.42	5.77	BARCSOYSSR_07_0266	BARCSOYSSR_07_0300	Ping et al. (2016)
18	*Rps12*	18 (G)	PI 399036	56	56.3	BARCSOYSSR_18_1840	Sat_064	Sahoo et al. (2017)
19	*Rps13*	18 (G)	PI 399036	-	-	Sat_064	BARCSOYSSR_18_1859	Sahoo et al. (2021)
20	*RpsUN1*	3 (N)	PI 567139B	3.2	4.3	Satt159 (BARCSOYSSR_03_0180)	BARCSOYSSR_03_0250	Lin et al. (2013), Li et al. (2016a)
21	*RpsUN2*	16 (J)	PI 567139B	36.9	37.3	BARCSOYSSR_16_1275	Sat_144 (BARCSOYSSR_16_1294	Lin et al. (2013), Li et al. (2016a)
22	*Rps Yu25*	3 (N)	Zheng 92116	3.19	3.33	Sat_186 (BARCSOYSSR_03_0204)	Satt_152	Sun et al. (2011)
23	*RpsYD29*	3 (N)	Yudou 29	3.9	4.1	SattWM82-50	Satt1k4b	Zhang et al. (2013b)
24	*RpsYD25*	3 (N)	Yudou 25	2.2	4.5	Satt1k3	BARCSOYSSR_03_0253	Fan et al. (2009), Zhong et al. (2020)
25	*RpsYB30*	19 (L)	Youbian 30	33.9	34.8	Satt497 (BARCSOYSSR_19_0760)	Satt313 (BARCSOYSSR_19_0788)	Zhendong et al. (2010)
26	*RpsSu*	10 (O)	Su88-M21	1	39.4	Satt358	Sat_242 (BARCSOYSSR_10_1104)	Wu et al. (2011b)
27	*RpsZS18*	2 (D1b)	Zaoshu18	43.37	44.3	ZCSSR33	ZCSSR46	Yao et al. (2010), Zhong et al. (2018a)
28	*RpsSN10*	13 (F)	Suinong 10	16.6	16.9	Satt423 (BARCSOYSSR_13_0264)	Satt149 (BARCSOYSSR_13_0245)	Yu et al. (2010)
29	*Rps1?*	3 (N)	Waseshiroge	3.9	4.5	Satt009 (BARCSOYSSR_03_0226)	T0003044871	Sugimoto et al. (2011)
30	*RpsJS*	18 (G)	Nannong 10–1	56.3	56.6	BARCSOYSSR_18_1859	BARCSOYSSR_18_1864	Sun et al. (2014a)
31	*RpsWY*	3 (N)	Wayao	2.9	3.4	Satt631 (BARCSOYSSR_03_0162)	Satt152 (BARCSOYSSR_03_0192)	Cheng et al. (2017)
32	*RpsQ*	3 (N)	Qichadou 1	3	3.1	BARCSOYSSR_03_0165	InDel281	Li et al. (2017a)
33	*RpsHN*	3 (N)	Meng8206	4.2	4.5	SSRSOYN-25	SSRSOYN-44	Niu et al. (2017)
34	*Rps HC18*	3 (N)	Huachun 18	4.5	4.6	BARCSOYSSR_03_0269	BARCSOYSSR_03_0272	Zhong et al. (2018b)
35	*RpsX*	3 (N)	Xiu94-11	2.9	3.2	InDelxz6	BARCSOYSSR_03_0175	Zhong et al. (2019)
36	*RpsGZ*	3 (N)	Guizao1	32.3	-	Gm_03_bin31	-	Jiang et al. (2020)
37	*-*	16 (J)	-		4.0*	-	BARC-014467–01559 -	Huang et al. (2016)
38	*-*	20 (I)	-	-	46.6*	BARC-013645–01207	-	Huang et al. (2016)

^a^ Physical position of the left marker and right flanking markers is based on the genome assembly Wms.82. v1. a2 and approximate physical positions with an asterisk (*) are based on the genome assembly Glyma. Wm82. a1.

^b^ Physical positions and associated markers on *Rps3a* are based on Gordon et al. (2007).

**TABLE 2:** Details of QDRL for *P. sojae* resistance identified through the bi-parental mapping approach in soybean.

^b^Plant material/population	QDRL/genomic region	^a^Potential linked marker	Chromosome	PVE/*R* ^2^ (%)	Marker	Environment	Reference
Conrad × Sloan (RILs)	2	Satt579 and Satt600; Satt252 and Satt149	2 and 13	10.6 and 32.4	SSR	Growth chamber	Burnham et al. (2003)
Conrad × Harosoy (RILs)	2	Satt266 and Satt579; Satt252 and Satt423	2 and 13	15.9–35.0	SSR	Growth chamber	Burnham et al. (2003)
Conrad × Williams (RILs)	2	Satt579 and Satt600; Satt252 and Satt149	2 and 13	20.7–21.4	SSR	Growth chamber	Burnham et al. (2003)
Conrad × OX760-6-1 (RILs)	1	Satt414 and Satt596	16	13.7–21.5	SSR	Field	Weng et al. (2007)
Conrad × OX760-6-1 (RILs)	3	OPL18 and Satt274; Satt509 and Satt030; Satt343 and OPG16600	2 and 13	2.4–21.6%	RAPDs and SSR	Greenhouse	Han et al. (2008)
Hefeng 25 × Conrad (RILs)	8	Satt579 and Sat_089; Satt325 and Satt343; Satt277 and Satt365	2.6, 8, 11, and 13	4.24–27.98	SSR	Greenhouse and Field	Li et al. (2010)
V71-370 × PI407162 (RILs)	3	Satt414, Satt529, Sat_163, and SLP142	16, 18, and 20	7–32	SSR	Greenhouse	Tucker et al. (2010)
Conrad × Sloan (RILs)	5	Satt353, Sct_033, Satt574, GMH_OSU31, GML_OSU10, and F424_294	12, 13, 14, 17, and 19	4–7	SSR; SNP	Greenhouse	Wang et al. (2010)
Su88-M21 × Xinyixiaoheidou (RILs)	3	Satt520, Satt557,Satt598, Satt651, Satt420, and Sat_274	6, 10, and 15	4.3–15.9	SSR	Greenhouse	Wu et al. (2011c)
Conrad × Sloan (RILs)	5	Satt527, BARCSOYSSR_19_1473, BARC-060037–16311, and BARCSOYSSR_18_1777	1, 18, and 19	4.8–19.6	SNP	Greenhouse	Wang et al. (2012)
S99–2,281 × PI 408105A (RILs)	2	Sat_154 Sat_375, Sat_300, and BARC-023721–03465	13 and 17	7.5–35.8	SSR; SNP	Greenhouse	Nguyen et al. (2012)
OX20–8 × PI 398841, (RILs)	3	BARC-044479–08708, BARCSOYSSR_13_1103, BARC-031343–07057, (BARCSOYSSR_13_0981), and BARCSOYSSR_13_1131	1, 13, and 18	4–16	SNP	Field	Lee et al. (2013a)
OX20–8 × PI 407861A (RILs)	9	BARC-051883–11286, Sat_234, and BARCSOYSSR_15_0160	3, 4, 8, 10, 13, 15, and 18	2.4–8.6	SNP	Greenhouse	Lee et al. (2013b)
Combined populations (6 NAM)	16	BARC-025777–05064, BARC-047665–10370, BARCSOYSSR13_1106 and BARCSOYSSR13_1103	1, 3, 12, 13, 16, and 18	4–45	SNP	Greenhouse amd field	Lee et al. (2014)
Conrad × Sloan, (RILs)	10	BARC_2.0_Gm18_56710850, BARC_2.0_Gm18_56876857, BARCSOYSSR_19_1286 and BARC_2.0_Gm19_46116996	1, 4, 9, 15, 16, 18, and 19	2–13.6	SNP	Greenhouse	Stasko et al. (2016)
PI 399036 × AR2 (AX20925) (RILs)	6	BARC-064609–18739, BARC-039977–07624, BARC-042881–08448 and BARC-019805–04379	2, 3, 6, 12, 15, and 19	5–14	SSR	Growth chamber	Abeysekara et al. (2016)
PI 399036 × AR3 (AX20931) (RILs)	7	BARC-065787–19749, BARC-056237–14178, BARC-017625–02635 and BARC-055533–13402	2, 7, 5, 8, 9, 13, 14, 15, 17, and 20	5–30	SSR	Growth chamber	Abeysekara et al. (2016)
Combined populations (2 NAM)	4	Gm13_29043806_T_C, Gm13_39560450_G_A, and Gm06_11776489_C_A	6, 13, and 18	7–42.2	SNP	Growth chamber	Scott et al. (2019)
PI 449459 × Misty	2	Chr13:28842184, Chr13:30776191, Chr19:50040258, and Chr19:50556102	13 and 19	13.1–17.6	SNP	Growth chamber	de Ronne et al. (2019)
Hefeng 25 ×DongongL-28	2	Chr03-41803925, Chr03-41822143, Chr03-3904775, and Chr03-4404630	3	5.8–56.0	SNP and SLAF	Growth chamber	Zhao et al. (2020)
Williams × PI 407974B and Williams × PI 424487B	3	ss715586321, ss715632438, and ss715632427	3 and 18	56–89	SSR and SNP	-	Bolanos-Carri et al. (2021)

^a^ Markers which explained maximum phenotypic variations and near identified genomic regions.

^b^ PRSR, resistant parent depicted in bold letters.

## Genetics of complete resistance versus partial resistance

There are two types of resistance to *P. sojae* reported in soybean, namely, complete resistance and partial resistance (Sugimoto et al., 2012). Complete resistance is race-specific and exhibits a single dominant resistance gene (*Rps*) that provides immunity or near immunity, whereas partial resistance is controlled by major and minor genes, and it restricts pathogen colonization and spread (Dorrance et al., 2003, 2004; Sugimoto et al., 2012). Previous studies over the last 2–3 decades identified both complete and partial resistance to *P. sojae* (Burnham et al., 2003; Dorrance et al., 2004; Sugimoto et al., 2012; Jang and Lee 2020).

During the single dominant gene resistance mechanism against *P. sojae*, expressed products of *Rps* genes interact with those of *P. sojae* through a gene-for-gene interaction and prevent disease development in plants (Hartwig et al., 1968; Schmitthenner, 1999; Burnham et al., 2003; Fan et al., 2009). There are very few reports explaining the detailed expressed products of *Rps* genes; Gao et al. (2005) mentioned the role of coiled-coil–nucleotide-binding–leucine-rich repeat (CC-NB-LRR)-type proteins in the case of *Rps1-*k locus ,and Li et al. (2021) demonstrated that *E3 ligase GmPUB1* protein is required for the interaction of *P*. *sojae* effector protein *Avr1b* with the resistance of *Rps1b* and *Rps1k* in soybean. As an example, during an investigation of the inheritance pattern of *Rps* genes, Li et al. (2017b) used detached-petiole and hypocotyl inoculation methods in F_2_ and F_2:3_ populations derived from a cross “Zhonghuang47” × “Xiu94-11.” A segregation ratio of 3:1 for the resistance and the susceptible reaction indicated a single dominant gene for *P*. *sojae* resistance in their study. All the *Rps* genes provide race-specific and complete resistance with the exception of *Rps2*, which provides incomplete resistance (Mideros et al., 2007).

### Genes for complete resistance

**FIGURE 1:**
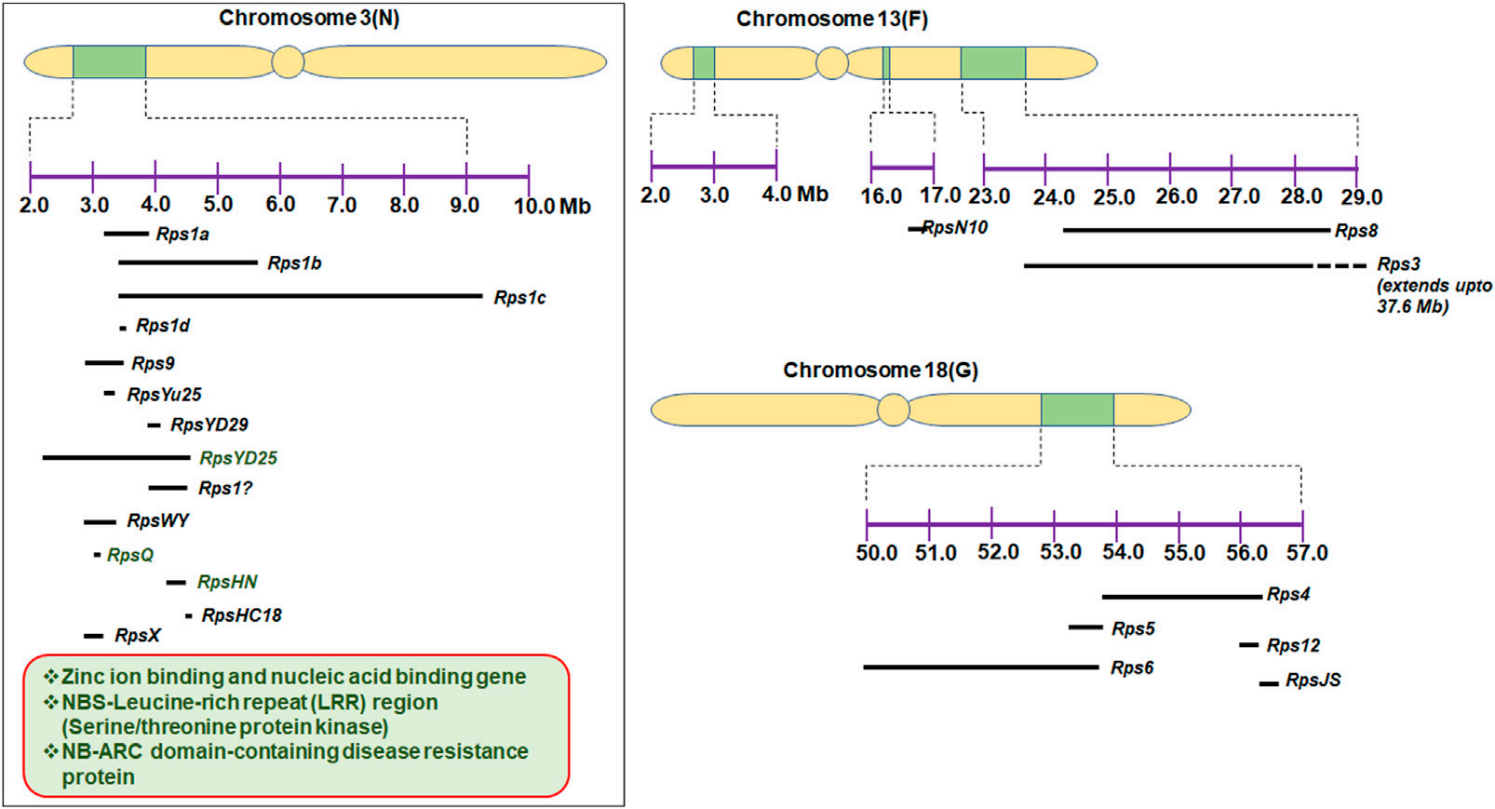
Genomic regions of chromosomes 3, 13, and 18, where more than 20 *Rps* genes were mapped; some potential characterized candidate genes are also depicted on chromosome 3.

To the best of our knowledge, more than 30 *Rps* genes/alleles have been reported and are present on 10 different chromosomes in soybean (Table 1). Most of the *Rps* loci are located on chromosome 3 (14 genes), followed by chromosome 18 (6 genes) and chromosome 13 (5 genes). The *Rps* genes on these three chromosomes constitute approximately 70% of the total *Rps* genes reported (Figure 1). *Rps1* (with five different alleles, *Rps1a*, *Rps1b*, *Rps1c*, *Rps1d*, and *Rps1k*), *Rps7*, *Rps9*, *RpsYu25*, *RpsYD29*, *RpsYD25*, *RpsUN1*, *RpsWY*, *RpsQ*, *RpsHC18, RpsX*, *RpsHN*, and *RpsGZ* and an unnamed *Rps* gene (*Rps1?*) were mapped on the short arm of chromosome 3 (Figure 1; Table 1). Similarly, *Rps4*, *Rps5*, *Rps6*, *Rps12*, *Rps13*, and *RpsJS* are located on chromosome 18; *Rps2*, *RpsUN2*, and one unknown *Rps* are located on chromosome 16; *Rps3* (three alleles, *Rps3a*, *Rps3b*, and *Rps3c*) and *RpsSN10* which was linked with *Rps8* were mapped on chromosome 13. Furthermore, the remaining genes, namely, *RpsZS18*, *Rps11*, *RpsSu*, *Rps10*, and *RpsYB30*, and an unnamed *Rps* were identified on chromosomes 2, 7, 10, 17, 19, and 20, respectively (Sandhu et al., 2004; Sandhu et al., 2005; Gordon et al., 2007; Yao et al., 2010; Yu et al., 2010; Wu et al., 2011b; Zhang et al., 2013a; Lin et al., 2013; Sun et al., 2014a; Li et al., 2016a; Huang et al., 2016; Ping et al., 2016; Sahoo et al., 2017; Sahoo et al., 2021). A few genomic regions were repeatedly found in many mapping studies using bi-parent populations. For example, on chromosome 3, a genomic region of ∼2 Mb was found to be a hot spot, where major resistance was identified in over 10 investigations using different resistance sources (Figure 1). Zhong et al. (2019) identified *RpsX* in soybean cultivar Xiu94-11; subsequently, it was revealed that *RpsX* was located in the 242-kb genomic region spanning the *RpsQ* locus on chromosome 3. Zhong et al. (2020) fine-mapped *RpsYD25* in 1127 F_3:4_ families derived from “Zaoshu18” and “Yudou25;” subsequently, 7 out of 178 soybean genotypes containing *RpsYD25* were identified using five co-segregated SSR markers. Recently, Jiang et al. (2020) have fine-mapped *RpsGZ* to a 367.371-kb genomic region on chromosome 3 in recombinant inbred lines (RILs) derived from a cross of the resistant cultivar “Guizao1” and the susceptible cultivar “BRSMG68.” Sahoo et al. (2017) identified *Rps12* on chromosome 18 in an RIL population developed by crossing the *P*. *sojae* resistant cultivar “PI399036” with the susceptible “AR2” line, and this gene was mapped at 2.2 cM proximal to the *NBSRps4/6*-like sequence that co-segregated with the *Phytophthora* resistance genes *Rps4* and *Rps6*.

In general, *Rps* gene efficacy is limited to 8–15 years (Grau et al., 2004; Sugimoto et al., 2012). Therefore, continuous efforts are required for the identification of new *Rps* genes and for the development of PRSR-tolerant cultivars. Under the conditions of high disease pressure, cultivars with complete resistance are far more effective than those having partial resistance to *P. sojae* (Schmitthenner, 1999; Dorrance et al., 2003). Contradictorily, partial resistance conferred by many QDRL has been found to be durable compared to complete resistance (single *Rps* gene) in the United States where *P. sojae* races evolve at a much faster rate to knock down even the most effective *Rps* genes (Dorrance et al., 2003). This indicates the significance of both complete resistance and partial resistance to *P. sojae* in different situations.

### Quantitative disease resistance loci for partial resistance

Partial resistance to *P. sojae* is a quantitative trait which is usually race non-specific and provides long-term resistance stability against the pathogen (Schmitthenner 1985; Dorrance et al., 2003; Dorrance et al., 2008; Wang et al., 2012). Partial resistance has moderate to high heritability and thus can be improved through selection pressure. For stable and durable management of PRSR, partial resistance along with complete resistance (*Rps* genes) may be used, as both types of resistance have different mechanisms to respond to PRSR.

Usually, the levels of partial resistance are evaluated using lesion length measurement, root rot score, tray test, inoculum layer test, or field evaluation (Tooley and Grau 1984; Dorrance et al., 2008). The development of cultivars with increased levels of partial or incomplete resistance needs the identification and characterization of novel resources of partial resistance.

**FIGURE 2:**
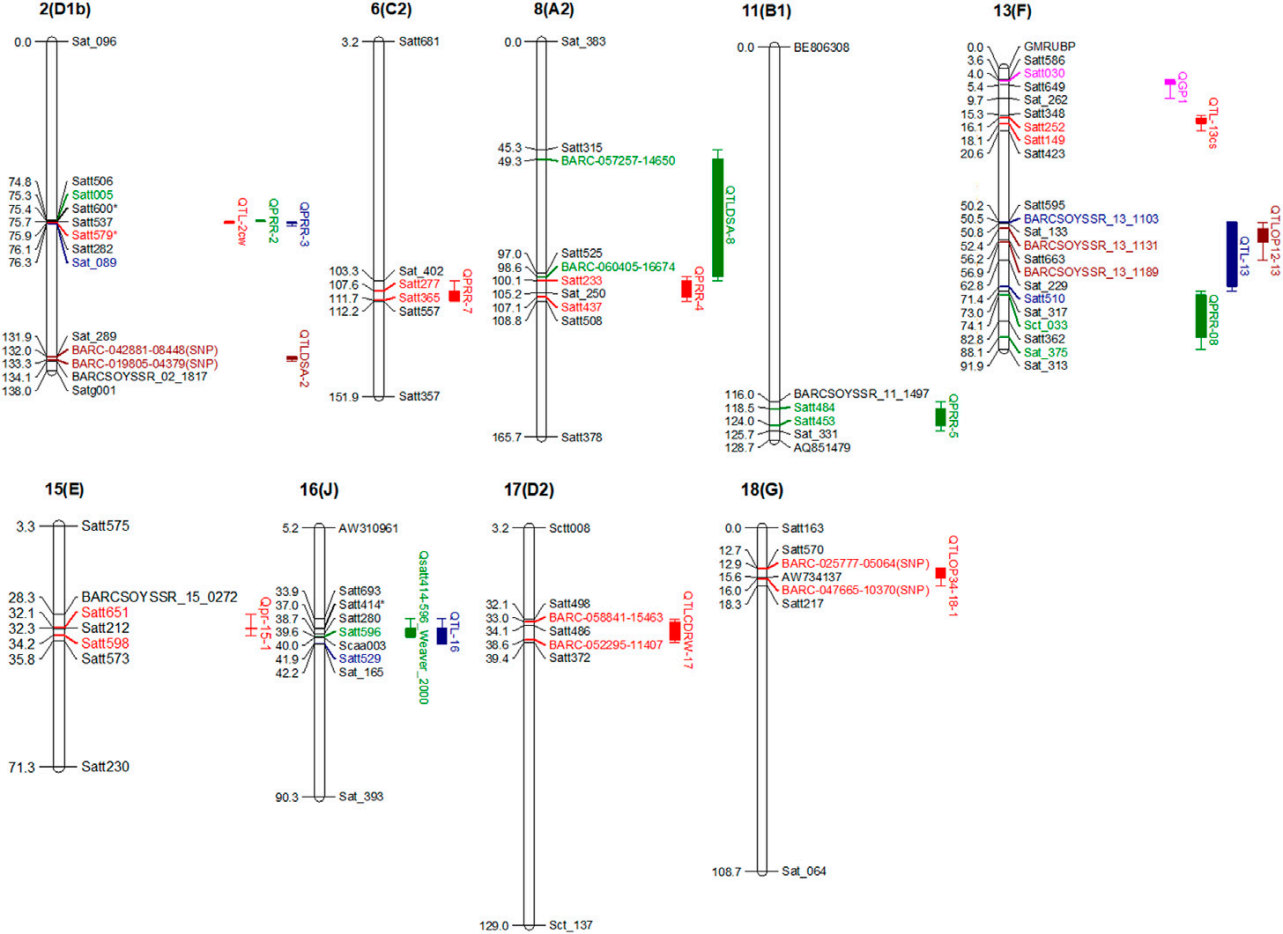
Major QDRL (phenotypic variations explaining (PVE) more than 10%) identified for *P. sojae* resistance along with their flanking makers and chromosomal locations.

Partial resistance or field resistance to *P. sojae* is governed by several genomic regions called quantitative trait loci (QTL or alternatively termed QDRL), each contributing a certain magnitude of resistance (Scott et al., 2019). There are a number of resources that have been utilized for mapping QDRL for partial resistance to *P. sojae* (Table 2). Extensive mapping studies using two contrasting parents in soybean reported about more than 90 QDRL for partial resistance to *P. sojae* (Table 2; Figure 2). Later on, the large confidence interval spanning genomic regions against *P. sojae* was further narrowed down through fine-mapping to pinpoint the exact position of QDRL (Huang et al., 2016; Karhoff et al., 2019). The cultivar “Conrad” that does not exhibit *Rps* genes but shows high partial resistance has been extensively used in QDRL mapping, identifying over 35 QDRL using different bi-parental populations (Burnham et al., 2003; Weng et al., 2007; Han et al., 2008; Li et al., 2010; Wang et al., 2010; Wang et al., 2012; Stasko et al., 2016). Some common QDRL were identified in a “Conrad” × “Sloan” population against three isolates of *P. sojae*, showing that a common resistance mechanism may occur in response to the individual inoculated isolates (Stasko et al., 2016). The detailed list of recent mapping studies using bi-parental populations leading to the identification of major QDRL along with significant markers imparting partial resistance to *P. sojae* is given in Table 2. Although over 15 QDRL explained more than 10% phenotypic variance (PV), the majority of QDRL explained <10% of the PV for partial resistance toward PRSR (Table 2) (Burnham et al., 2003; Weng et al., 2007; Han et al., 2008; Li et al., 2010; Nguyen et al., 2012; Lee et al., 2013a; Lee et al., 2014; Abeysekara et al., 2016; de Ronne et al., 2019; Scott et al., 2019). Apart from RILs, nested association mapping (NAM) populations have also been used to map QDRL associated with PRSR (Lee et al., 2014). Recently, Scott et al. (2019) have carried out inoculation of two Soy-NAM populations with *P. sojae* isolate Win371 for the identification of major QDRL (Figure 2). Four major QDRL were identified by Abeysekara et al. (2016) using RILs derived from “AX20925” (PI 399036 × AR2) and “AX20931” (PI 399036 × AR3). In the latest study, Zhao et al. (2020) identified quantitative trait nucleotides (QTNs) explaining up to 56% PV on chromosome 3 using RILs derived from crossing “DongnongL-28” and “Hefeng 25.”

Alternatively, another approach, genome-wide association studies (GWAS), provide high-resolution mapping than the traditional bi-parental mapping strategy. In a SSR-based association mapping study among 214 soybean accessions, four SSR alleles, *viz.*, *Satt634-133*, *Satt634-149*, *Sat_222-168*, and *Satt301-190*, were found to be significantly associated with *P. sojae* partial resistance (Sun et al., 2014b). Similarly, in another GWAS on resistance to 11 *P. sojae* isolates involving 224 germplasm accessions, Huang et al. (2016) identified 14 marker–trait associations for PRSR resistance including five novel loci. In USDA soybean germplasm, significant associations were detected for 28 SNPs located on chromosomes 3, 13, and 18 (Chang et al., 2016). The updated information on all GWAS conducted on soybean against PRSR is given in Table 3. The majority of association studies identified SNPs explaining small variations (minor QDRL); however, some of the studies (Ludke et al., 2019; Rolling et al., 2020; Zhao et al., 2020) identified major QDRL explaining variations for PRSR. Ludke et al. (2019) conducted a SNP-based GWAS on 169 soybean cultivars for *P. sojae* resistance and identified four QDRL on two chromosomes (two each on chromosomes 3 and 15). Interestingly, the identified genomic regions were found to be co-localized with already known and annotated resistance genes. Recently, Rolling et al. (2020) analyzed QDRL in 478 and 495 plant introductions (PIs) against *P*. *sojae* isolates OH.121 and C2.S1, respectively, and 24 significant associated SNPs were identified. Five QDRL identified in this study were found to be co-localized with *P*. *sojae* meta-QDRL identified from previous bi-parental mapping studies (Rolling et al., 2020). Using available disease phenotypic information, Van et al. (2020) identified 75 novel QTNs using 16 panels consisting of 2,233 soybean accessions. The identified SNPs linked to QDRL can be used in marker-assisted selection for introgression and stacking of partial PRSR resistance loci for imparting durable resistance.

**TABLE 3:** Details of genomic regions associated with *P. sojae* resistance identified through the association mapping/GWAS approach in soybean.

No. of genotypes	GWAS loci	Chromosome	PVE (%)	Markers used	Method	References
214	4	2.17	5.24–8.14	138 SSRs	GLM and MLM	Sun et al. (2014b)
797	16	3, 13, and 19	2.5–3.8	19,303 SNPs	MLM	Schneider et al. (2016)
224	14	3, 6, 4, 9, 11, 15, 16, and 20	-	1,645 SNPs	GLM and MLM	Huang et al. (2016)
44–7431	28	3, 13, and 18	-	42,449	MLM	Chang et al. (2016)
189	32	3, 4, 5, 7, 10, 13, 14, and 18	-	33,625 SNPs	GML and MLM-Q + K	Qin et al. (2017)
337	26	1	6.14–11.18	60,862 SNPs	GML and MLM-Q + K	Niu et al. (2018)
279	3	13	-	59,845 SNPs	GLM and MLM (Q + K)	Li et al. (2016b)
169	8	3, 15	13.9–21.1	3,807 SNPs	MLM	Ludke et al. (2019)
478	24	2, 3, 5, 6, 10, 11, 12, 13, 18, and 20	2.28–12.1	34,248 SNPs	MLMM	Rolling et al., 2020
495	24	2, 3, 4, 5, 8, 11, 13, 14, 15, 16, 17, and 18	0.21–13.11	33,234 SNPs	MLMM	Rolling et al., 2020
225	8	3, 7, 14, 15, and 17	25.3–33.6	28,722 SNPs	CMLM and FARMCPU	Zhao et al. (2020)
2,233 (16 panels)	75	All chromosomes	-	∼33,641–40,954 SNPs	CMLM, MLMM_cof, and FARMCPU	Van et al. (2020)

^a^ Markers which explained maximum phenotypic variations and near identified genomic regions.

## Candidate genes for *Phytophthora* root and stem rot resistance

Characterization of putative genes imparting resistance to *P. sojae* has also been progressed. Graham et al. (2002) characterized the sequence of the *Rps2* genomic region. *Rps2* locus sequences included 16 resistance gene homologs with similarities to the *TIR/NBD/LRR* family of disease resistance genes, a leucine zipper protein, four gene sequences with similarities to Ca^2+^-binding domains of a *calmodulin* gene, and three genes with homology to an NtPRp27-like protein (Graham et al., 2002). Sequencing of the *Rps1k* locus identified a coiled-coil–nucleotide-binding site–leucine-rich repeat (*CC-NBS-LRR*)-type gene (Bhattacharyya et al., 2005). Further characterization of *Rps1k* by bacterial artificial chromosome (BAC) sequencing revealed the presence of two nucleotide-binding site–leucine-rich repeat (NBS-LRR)-encoding genes (*Rps1k-1* and *Rps1k-2*) (Gao et al., 2005; Gao and Bhattacharyya, 2008; Sandhu et al., 2009). Sandhu et al. (2004) demonstrated that the deletion of *NBSRps4/6* in mutant M1 is correlated with the loss of *Rps4* function. With the availability of a complete reference genome sequence, genomic regions of different *Rps* regions were analyzed for the identification of candidate resistance genes. A list of putative candidate genes for *P. sojae* resistance is given in Table 4 along with their gene annotations. The maximum number of candidate genes reported are from chromosome 3 (Zhang et al., 2013a; Zhang et al., 2013b; Sun et al., 2014a; Li et al., 2017a; Cheng et al., 2017; Niu et al., 2017; Zhong et al., 2018a; Zhong et al., 2019; Jiang et al., 2020; Zhong et al., 2020)*.* Some of these genes, *viz*., zinc ion binding- and nucleic acid-binding genes, NB-ARC domain-containing disease resistance proteins, and *NBS-LRR* genes, were functionally analyzed (Li et al., 2017a; Niu et al., 2017; Zhong et al., 2020) (Figure 1). Li et al. (2016a) reported multiple copies of *R-gene*-type annotation in *RpsUN1* and *UN2*. Li et al. (2016b) conducted GWAS in an association panel of 279 accessions and identified seven candidate genes on chromosome 13 that are reported to govern natural variations for partial resistance to *P. sojae*. Unlike Li et al. (2016a), *non-NBS-LRR* types of genes have also been proposed as candidates for another *Rps* allele on chromosome 3 (Cheng et al., 2017). Cheng et al. (2017) identified candidate genes against *P. sojae* using the high-throughput genome-wide sequencing approach by mapping 3,469 recombination bins in RILs. This study revealed the localization of *RpsWY* gene in bin 401 (on chromosome 3). Bin 401 was found to contain three genes, namely, *pentatricopeptide repeat-containing protein, transposase/serine/threonine protein*, and *non-specific lipid-transfer protein 3-like protein.* Sahoo et al. (2017) also reported several *NBS-LRR-like* genes in genetic investigations of *Rps12.* Jiang et al. (2020) and Zhong et al. (2020) also reported *NBS-LRR* and *zinc ion-binding* genes as candidate genes by fine mapping of *RpsYD25* and *RpsGZ.* Though reference genome sequencing can provide information on the majority of genes present in the identified genomic region, *de novo* sequencing of the haplotype carrying the target *Rps* gene is important to identify candidate genes.

**TABLE 4:** Putative candidate genes identified for *P. sojae* resistance.

Parents	Rps genes	Gene model number	Gene function	References
Conrad × Sloan	*-*	*Glyma19g40800*	Transducin/WD40 domain-containing	Wang et al. (2012)
		*Glyma19g40840*	Pectinesterase	
		*Glyma19g40940*	Glycoside hydrolase family 28 protein	
		*Glyma19g41590*	2-Deoxyglucose-6-phosphate phosphatase	
		*Glyma19g41900*	Phloem-specific lectin PP2-like protein	
		*Glyma19g42120*	Heparan-alpha-glucosaminide N-acetyltransferase	
		*Glyma19g42200*	Rapid alkalinization factor	
		*Glyma19g42210*	Rad9	
		*Glyma19g42220*	Respiratory burst oxidase 2	
		*Glyma19g42240*	Histone H2A 7	
		*Glyma19g42390*	Cyclin-dependent protein kinase	
Wandou 15 and Williams	*Rps10*	*Glyma17g28950.1*	Serine/threonine (Ser/Thr) protein kinases	Zhang et al. (2013a)
		*Glyma17g28970.1*	Serine/threonine (Ser/Thr) protein kinases	
Jikedou 2 × Yudou 29	*RpsYD29*	*Glyma03g04030.1*	NBS-LRR	Zhang et al. (2013b), Gao and Bhattacharyya (2008)
		*Glyma03g04080.1*	NBS-LRR	
Nannong 10–1 (P1) × 06–070583 (P2)	*Rps JS*	*Glyma18g51930*	NBS-LRR	Sun et al. (2014a)
		*Glyma18g51950*	NBS-LRR	
		*Glyma18g51960*	NBS-LRR	
Germplasm panel (797)	*-*	*Glyma.03G034400*	NBR-gene	Schneider et al. (2016)
		*Glyma.03G034200*	Plant defense	
		*Glyma.03G035700*	Abscisic acid responsive stress	
		*Glyma.13G194100*	NB-LRR-encoding genes	
		*Glyma.19G245400*	PR4-related chitin-binding proteins	
		*Glyma.19G248900*	Ethylene/JA responsive transcription factor	
Germplasm panel (279)	*-*	*Glyma13g32980*	*Coat protein I* (COPI)-related gene	Li et al. (2016b)
		*Glyma13g33900*	2OGFE (II) oxygenase family protein	
		*Glyma13g33512*	Pentatricopeptide (PPR) repeat	
		*Glyma13g33536*	Leucine-rich repeat domain protein	
		*Glyma13g33740*	Leucine-rich repeat domain protein	
		*Glyma13g33243*	Gpi16 subunit	
		*Glyma13g33260*	Zn-finger protein	
Germplasm panel (224)	*-*	*Glyma15g41680*	LEM3 (ligand-effect modulator 3) family/CDC50-related	Huang et al. (2016)
		*Glyma03g28660*	ARF-related/ADP-ribosylation factor	
		*Glyma16g30140*	Predicted lipase class 3 gene	
		*Glyma16g04700*	Thioredoxin	
		*Glyma20g39240*	DEAD/DEAH box helicase	
		*Glyma06g01080*	2OG-Fe (II) oxygenase superfamily	
		*Glyma16g14080*	Serine/threonine protein kinase	
		*Glyma11g11100*	Phototropic-responsive NPH3 family protein	
		*Glyma16g31930*	Zinc finger domain	
		*Glyma03g04960*	Lipid transport protein	
		*Glyma04g40800*	Serine/threonine protein kinase	
		*Glyma09g04310*	Ankyrin repeat and calmodulin-binding motif	
Germplasm panel (189)	*-*	*Glyma.03g034400*	LRR and NB-ARC domains containing disease resistance protein	Qin et al. (2017)
		*Glyma.05g209300*	Disease resistance protein (TIR-NBS class)	
		*Glyma.05g213400*	Disease resistance responsive (dirigent-like protein) family protein	
		*Glyma.13g184800*	LRR and NB-ARC domains containing disease resistance protein	
		*Glyma.07g007800*	Disease resistance protein RPS4-RELATED	
		*Glyma.03g037000*	LRR and NB-ARC domains containing disease resistance protein	
		*Glyma.04g205200*	Defense response	
		*Glyma.13g028100*	RPS4-related disease resistance protein	
		*Glyma.03g149600*	Resistance to Phytophthora 1	
		*Glyma.10g127500*	Disease resistance responsive (dirigent-like protein) family protein	
		*Glyma.10g129400*	Disease resistance family protein/LRR family protein	
		*Glyma.10g184300*	RPS4-related disease resistance protein	
		*Glyma.10g196700*	Disease resistance protein (CC-NBS-LRR class) family	
		*Glyma.14g079500*	Arabidopsis broad-spectrum mildew resistance protein RPW8	
		*Glyma.14g079600*	Arabidopsis broad-spectrum mildew resistance protein RPW8	
Jikedou 2 × Qichadou 1	*RpsQ*	*Glyma.03g27200*	Protein with a typical serine/threonine protein kinase structure	Li et al. (2017a)
Meng8206 × Linhedafenqing and Meng8206 Zhengyang	*RpsHN*	*Glyma.03g04260*	NB-ARC domain-containing disease resistance protein	Niu et al. (2017)
		*Glyma.03g04300*	NB-ARC domain-containing disease resistance protein	
		*Glyma.03g04340*	Serine/threonine protein kinase	
Huachun 2 ×Wayao	*RpsWY*	*Glyma03g04350*	Pentatricopeptide repeat-containing protein	Cheng et al. (2017)
		*Glyma03g04360*	Transposase/serine/threonine protein	
		*Glyma03g04370*	Non-specific lipid-transfer protein 3-like protein	
Germplasm panel (337)	*-*	*Glyma01g32800*	Serine/threonine protein kinase	Niu et al. (2018)
		*Glyma01g32855*	Leucine-rich repeat protein kinase family proteins	
Williams×Zaoshu18	*RpsZS18*	*Glyma.02g245700*	EF-hand calcium-binding domain	Zhong et al. (2018a)
		*Glyma.02g245800*	pfkB carbohydrate kinase	
Germplasm panel (169)		*Glyma03g03480*	Auxin-responsive family protein	Ludke et al. (2019)
		*Glyma03g04990*	Aalanine-glyoxylate aminotransferase/beta- Alanine-pyruvate aminotransferase	
		*Glyma03g05070*	Short-chain dehydrogenase/reductase (SDR) family protein	
		*Glyma15g20550*	Pectinesterase family protein	
		*Glyma15g21130*	Expansin-like B3 precursor (EXLB3)	
Zhonghuang47 × Xiu94-11	*RpsX*	*Glyma.03g027200*	Leucine-rich repeat (LRR) region	Zhong et al. (2019)
Hefeng 25 × DongongL-28; Germplasm (225)	*-*	*Glyma.03G033700*	C2H2-like zinc finger protein	Zhao et al. (2020)
		*Glyma.03G033800*	Cell wall β-expansin protein	
Germplasm panel (376)	*-*	*Glyma05g146400*	Mannosyl oligosaccharide glucosidases	Van et al. (2020)
		*Glym05g146500*	Mannosyl oligosaccharide glucosidases	
		*Glym.05g146600*	ER metallopeptidase	
		*Glyma05g146900*	Heparan sulfate glycosyltransferase-related	
PI 449459 × Misty	*-*	*Glyma.13G190400*	NBS-LRR	de Ronee et al. (2019)
		*Glyma.19G262700*	AP2/ERF-type transcription factor	
Zaoshu18 × Yudou25	*RpsYD25*	*Glyma.03g034700*	Zinc ion binding- and nucleic acid-binding gene	Zhong et al. (2020)
		*Glyma.03g034800*	NBS-LRR	
		*Glyma.03g034900*	NBS-LRR	
Guizao1 × BRSMG68	*RpsGZ*	*Glyma.03G034400*	Disease resistance protein (NBS-LRR class), putative	Jiang et al. (2020)
		*Glyma.03G034500*	Disease resistance protein (NBS-LRR class), putative	
		*Glyma.03G034800*	Disease resistance protein (NBS-LRR class), putative	
		*Glyma.03G034900*	Disease resistance protein (NBS-LRR class), putative	
		*Glyma.03G035000*	Domain of unknown function DUF223	
		*Glyma.03G035100*	PIF1-like helicase	
		*Glyma.03G035200*	CW-type zinc finger; B3 DNA-binding domain	
		*Glyma.03G035300*	Disease resistance protein (NBS-LRR class)	
		*Glyma.03G035400*	PPR repeat	
		*Glyma.03G035500*	Plant mobile domain	
		*Glyma.03G035600*	Protease inhibitor/seed storage/LTP family	
		*Glyma.03G035800*	Pollen allergen; rare lipoprotein A (RlpA)-like double-psi beta-barrel	
		*Glyma.03G035900*	Membrane attack complex/perforin domain	
		*Glyma.03G036000*	Protein tyrosine kinase; serine–threonine protein kinase	
		*Glyma.03G036200*	Multidrug resistance protein	

*Rps 11* showed resistance to 12 races of *P. sojae*; therefore, it is a broad-spectrum resistance gene (Wang et al., 2021). Wang et al. (2021) sequenced the genome of “PI 594527” by long-read sequencing, and the assembled genome sequence identified that the *Rps11* locus was present in a genomic region harboring a cluster of 12 *NLR* genes of a single origin in soybean. Fine mapping and gene expression analysis pinpointed a 27.7-kb *NLR* gene (Wang et al., 2021). Genetic transformation of an *Rps11-*coding DNA sequence in a susceptible soybean genotype conferred a resistant phenotype. Pan-genome analysis revealed that *Rps11* is a unique gene in “PI 594527” and does not have any other allelic copy in the other genotypes. The isolation of *Rps11* will help soybean breeders accelerate the improvement of broad-spectrum resistance to *P. sojae* in soybean. The unique structural features of *Rps11* make it a suitable model to investigate the resistance mechanism to further improve high-yielding cultivars.

## Transcriptomic studies on PRSR

Recent developments in the genomics of *P. sojae* and soybean have made this pathosystem a model to understand molecular bases underpinning plant–oomycete interactions (Guo et al., 2011). Furthermore, transcriptomics of PRSR resistance in soybean has been extensively carried out to study the candidate genes and the role of biochemical pathways involved in conferring resistance. Through microarray analysis, genes governing pathogenesis-related proteins and enzymes involved in phytoalexin biosynthesis were found to be upregulated and reached a peak at 24 dpi. On the other hand, genes encoding lipoxygenases and peroxidases were found to be downregulated during the infection process (Moy et al., 2004).

To gain deep insights into the molecular basis of resistance to *P. sojae*, differential gene expression in response to *P. sojae* infection in the cultivar “Suinong 10” was studied by Xu et al. (2012). A total of eight transcripts were found to be upregulated in the treated plants as compared to those of the control. These transcripts are responsible for enzymes involved in the phytoalexin biosynthesis pathway and pathogenesis-related proteins and some defense response-related proteins such as phenylalanine ammonia-lyase, WRKY transcription factor 31, isoflavone reductase, pleiotropic drug resistance protein 12, and major allergen *Pru ar 1* (Xu et al., 2012). Molecular responses induced by different *Rps* genes and the association of phytohormone signaling pathways with disease reactions to *P. sojae* infection were studied by Lin et al. (2014). Transcriptome analysis on 10 near-isogenic lines (NILs) (*Rps1-a*, *1-b*, *1-c*, *1-k*, *Rps3-a*, *3-b*, *3-c*, *Rps4*, *5*, and *6*, each in the genetic background of “Williams”) and the susceptible genotype, “Williams” during pre- and post-inoculation was carried out to identify differentially expressed genes (DEGs) across different treatments (Lin et al., 2014). A total of 5,806 incompatible interaction genes (IIGs) were identified by comparing DEGs between “Williams” and NILs, and 1,139 compatible interaction genes (CIGs) were identified in “Williams.” Of these 5,806 IIGs, 23 were found to be common across 10 NILs and are mostly associated with biotic and abiotic stress responses, suggesting the overlap of molecular responses induced by different *Rps* genes. Two *NPR-1-*like IIGs, *Glyma02g45260* and *Glyma14g03510*, were involved in mediating the SA signaling pathway during an incompatible reaction, suggesting the role of the SA pathway in genetic resistance. Several *JAZ*-like proteins that repress the jasmonic acid (JA) pathway were found, such as IIGs and/or CIGs. These proteins were downregulated in NILs and were upregulated in “Williams.” Also, a *JAR1* homolog, *Glyma07g06370* that activates the JA signaling pathway, was upregulated during the susceptible reaction in Williams. Genes that repress the ethylene (ET) pathway were found to be downregulated in NILs and upregulated in “Williams,” suggesting that the ET pathway was repressed during the susceptible host reaction in “Williams” and activated in NILs during the incompatible reaction. In addition, three *BAK1* homolog IIGs that activate brassinosteroid (BR) signaling were found to be upregulated in NILs, suggesting the role of the BR signaling pathway during defense against *P. sojae.*

## Role of transcription factors

Transcription factors (TFs) are master switches for regulating the expression of genes and controlling several signaling pathways (Chattopadhyay et al., 2019) and also play a vital role in different defense mechanisms in different plant species against different phytopathogens. In soybean, several TFs have been identified for their role in regulating genes and pathways involved in resistance to *P. sojae*. A *bHLH* (basic helix–loop-helix) transcription factor associated with resistance to *P. sojae* was functionally characterized through its hypo- and hyper-expression in a resistant soybean genotype, “L77-1863,” and was designated as *GmPIB1*. *GmPIB1* represses the expression of the *GmSPOD1* gene by directly binding to its promoter. Through RNAi assay, it was found that *GmSPOD1* is involved in the production of reactive oxygen species (ROS) during *P. sojae* infection. Hence, the role of *GmPIB1* TF in *P. sojae* resistance through reduced ROS production has been established (Cheng et al., 2018). Several *ethylene-responsive element binding factor* (*ERF*) transcription factors are linked with disease resistance in different plants (Gu et al., 2000; Song et al., 2005). An ERF-associated amphiphilic repression (EAR) motif-containing ERF TF, *GmERF5*, conferring resistance to *P. sojae* through the positive regulation of pathogenesis-related (PR) genes, *PR10*, *PR1-1*, and *PR10-1*, has been identified (Dong et al., 2015). A TF gene, *GmWRKY40*, was found to impart resistance in soybean to *P. sojae* and acts as a positive regulator of ROS accumulation and the JA signaling pathway (Cui et al., 2019). A transcription factor, *GmMYB29A2*, was found to impart resistance to *P. sojae* infection in soybean through the regulation of *glyceollin I* accumulation (Jahan et al., 2020). *WRKY transcription factor 31* identified in response to *P. sojae* infection (Xu et al., 2012) was functionally characterized through overexpression and RNAi silencing (Fan et al., 2017). Gene *GmWRKY31* interacts with *GmHDL56* and jointly engages in the activation of *GmNPR1*, which in turn manifests resistance during the Suinong 10–*P. sojae* interaction. Another TF, *GMERF113*, was isolated from “Suinong 10” and characterized for its response to *P. sojae* infection in a susceptible genotype “Dongnog 5.” The overexpression of *GMERF113* in this genotype resulted in an enhanced resistance level and expression of pathogenesis-related genes, *PR1* and *PR10-1*. Thus, the role of *GMERF113* in the defense mechanism through positive regulation of these two pathogenesis-related genes has been well-demonstrated (Zhao et al., 2017).

## Role of enzymes and proteins

Fan et al. (2015) studied the expression of class 10 protein *Gly m 4l* and found its role in the resistance to *P. sojae.* Zhang et al. (2017) identified a *phenylalanine ammonia-lyase* (*PAL*) gene family member, *GmPAL2*.*1*, to be linked with resistance to *P. sojae* through reverse genetics. The role of enzyme class 4-coumarate: CoA ligase (4CL) in plant defense against pathogens has been investigated extensively (Ehlting et al., 1999). A member of the 4CL (enzyme 4-coumarate: CoA ligase) gene family, *GmPI4L*, identified in soybean is associated with resistance to *P. sojae* infection through the enhanced production of glyceollins, genistein, and daidzein in soybean, laying the foundation for the enzymatic basis for resistance to this pathogen (Chen et al., 2019). The mediator complex is a part of RNA polymerase II, which acts as a regulatory element of the transcription process. A mediator subunit, *GmMED16-1* in soybean, through its silencing, was found to govern *P. sojae* by modulating the transcription of *NPR1*, *PR1a*, and *PR5* genes (Xue et al., 2019).

## Role of miRNAs

MicroRNAs (miRNAs) are also known to be regulated under defense mechanisms in several plant species. Guo et al. (2011) revealed the role of miRNA in *P. sojae* resistance. Wong et al. (2014) identified miR393 and miR166, as triggered by heat-inactivated *P. sojae* hyphae, suggesting their roles in soybean basal defense. Knockdown of miR393 led to the increased susceptibility of soybean to *P. sojae.* The expression of iso-flavonoid synthesis genes was drastically reduced in miR393 knockdown roots, suggesting that miR393 promotes soybean defense against *P. sojae*.

## Molecular breeding for resistance to *P. sojae*

Soybean witnessed a significant improvement in yields over the past 60 years through conventional breeding approaches. Soybean yields were estimated to improve at the rate of 23 kg/ha/annum (Specht et al., 1999), and Wilcox (2001) reported an increase of 60% in seed yields over the past 60 years in the United States of America. The significant increase in yields has been witnessed mainly due to the toiling efforts of conventional breeding-based public sector soybean breeding programs. But considering the limitations of conventional breeding methods for *P. sojae* resistance improvement, further progress for yield enhancement is stagnated at the global level. The stagnated progress due to *P. sojae* infection can be further brought back to an accelerated track by the adoption of MAS and genomics-aided approaches in the PRSR resistance soybean breeding programs.

Molecular markers ranging from hybridization (RFLP and AFLP) and polymerase chain reaction-based markers (SSRs) to sequencing-based markers (SNPs) have been used to a greater extent for high-resolution mapping as well as for fine mapping of genomic regions governing the resistance to *P. sojae* (Table 2).

The identified major genomic regions for *P. sojae* resistance can be introgressed into elite soybean cultivars through the use of genomics-assisted breeding techniques, *viz.*, marker-assisted backcross breeding (MABB), marker-assisted recurrent selection (MARS), marker-assisted gene pyramiding (MAGP), and genomic selection (GS). The identified major QDRL can be targeted for introgression into elite cultivars using the MABB approach (Ribaut and Ragot, 2007; Choudhary et al., 2019). Selection of *Rps* gene for introgression is also very important as it depends on particular regions of cultivation. Dorrance et al. (2016) estimated pathotype variability in 11 different states of the US with 873 isolates and concluded that *Rps 6* and *Rps 8* are more effective against the majority of isolates collected from northern regions. Several efforts have been made for the introgression of single-gene (*Rps*)-mediated resistance into soybean cultivars for controlling PRSR (Roth et al., 2020). Six of these genes (*Rps1a*, *Rps1b*, *Rps1c*, *Rps1k*, *Rps3a*, *Rps 6*, and *Rps3a*) already exist in commercial varieties and provide disease management against *Phytophthora* root and stem rot (Roth et al., 2020), which were transferred with the help of conventional approaches. In Japan, “Hyogo Prefecture,” the black-seeded PRSR-resistant line, was used as the donor for introgression and for the development of resistant cultivars (Sugimoto et al., 2010). Although plant breeders use MAS-based approaches mainly for transferring *Rps* genes in soybean (Li et al., 2010; Ramalingam et al., 2020), due to high disease pressure, rapid evolution in the pathotypes of *P. sojae* has been witnessed over the past 3 decades, hence making vertical resistance ineffective. This forced the plant breeders to target partial resistance for the effective and sustainable management of PRSR (Schmitthenner, 1985). Studies on mapping QDRL dissected the genetic basis of partial resistance to *P. sojae* and revealed small-to-moderate effect QDRL, many of which individually explained less than 10% of phenotypic variance for PRSR in a population (Table 2). The difficulty of identifying small-effect QDRL in small mapping populations can be resolved by deploying joint linkage QDRL analysis of multiple populations (Lander and Kruglyak, 1995; Beavis 1998). Although relatively less, a good number of major QDRL have been mapped for PRSR partial resistance in soybean (Figure 2).

The utilization of MABB is restricted to the introgression of major QDRL only, which have more PVE (phenotypic variance explanation) percentages and limited localization in the genome, as it is very difficult to follow a large number of QDRL during introgression programs. Hence, other molecular breeding approaches such as MAGP, MARS, and genomic selection can serve as a good alternative for accumulating favorable QDRL (minor and major effects) for PRSR resistance. Pyramiding of PRSR-resistant QDRL was demonstrated by Li et al. (2010) by targeting seven consistent QDRL (detected across multiple environments) from two different donors (“Conrad” and “Hefeng 25”). Limited efforts of QDRL stacking for PRSR resistance revealed a significant increase in the tolerance level of introgressed lines, and the tolerance level against PRSR was found to be positively correlated with the number of QDRL stacked (Li et al., 2010). Recently, Karhoff et al. (2019) demonstrated the genetic gains from selections of a major QTL for partial resistance to *P. sojae.* The introgression of a resistance allele from the respective “PI 427105B” and “PI 427106” improved the genetic levels of resistance to *P. sojae* by ∼20% and ∼40%, respectively, and the yield by 13%–29% under diseased conditions (Karhoff et al., 2019). These are a few examples of PRSR resistance introgression through molecular breeding, demonstrating the fruitful results of genetic and genomic mapping for PRSR resistance. Dorrance et al. (2016) emphasized on stacking of *Rps* genes with strong partial resistance for limiting the loss caused by PRSR. With the new genomics-assisted breeding approaches, it will be practically more feasible and applicable in stacking of major genes for complete resistance and multiple QDRL of partial resistance for imparting sustainable PRSR resistance in soybean cultivars.

## Genome editing for understanding PRSR resistance

Not only naturally available and induced mutations are the source for introducing new resistance genes in crop improvement programs, but also genetic engineering and gene editing (genome editing) are techniques that enable precise and targeted modifications. Now, gene-editing technologies are gaining momentum for crop improvement as they are more similar to the widely accepted “mutation breeding” technology.

CRISPR/Cas9 gene editing is particularly useful in deciphering the plant–pathogen interaction and understanding effector-triggered immunity. Pathogen avirulence (*Avr*) effectors interplay with corresponding plant resistance (*R*) proteins and activate robust immune responses in the host plant. *Avr4/6*, an RxLR effector gene of *P. sojae*, which is recognized by soybean *R-*genes (*Rps6* and *Rps4*), was edited using CRISPR/Cas9 technology to study its possible role in pathogenicity (Fang and Tyler 2016). This study validated the contribution of *Avr4/6* in pathogen recognition by soybean *R-*gene loci, *Rps4* and *Rps6*. Ochola et al. (2020) engineered the promoter region of *PsAvr3b* gene which is recognized by *Rps3b*, and mutants with low *PsAvr3b* expression successfully colonized soybean plants carrying the cognate *R-*gene *Rps3b.* Wang L et al. (2020) edited *PsSu(z)12* gene associated with effector locus *Avr1b. PsSu(z)12* is epigenetically governed and encodes a core subunit of the *H3K27me3 methyltransferase complex.* CRISPR/Cas9-mediated H3K27me3 depletion within the *Avr1b* genomic region was correlated with impaired *Avr1b* gene silencing, and the mutants lost their ability to evade immune recognition by soybeans carrying *Rps1b* (Wang P et al., 2020). Tan et al. (2020) studied knockout mutants of *P. sojae* generated *via* the CRISPR/Cas9 system for the *PsGH7a* (*GH7 family cellobiohydrolase*) gene, and the mutants were found to have reduced virulence on susceptible soybean as compared to wild-type strain “P6497.” It is expected that in the future, the CRISPR/Cas9 system coupled with other genomic techniques will be an important approach to create disease-resistant cultivars that can withstand biotic stresses (Kumar et al., 2020).

## Challenges and future perspectives

The urgency and significance of *P. sojae-*resistant cultivar development can be realized from its vast spread and rapid occurrence of the disease across soybean-growing areas. This demands a strong emphasis on strengthening *P. sojae* resistance soybean breeding programs globally. Although significant progress has been made through the utilization of race-specific resistance genes (*Rps* genes), the rapid evolution of pathotypes in *P. sojae* resulted in resistance breakdown. This problem was quickly assessed by soybean breeders and, hence, shifted the focus to partial resistance (horizontal resistance) which provides relatively broad and highly durable resistance. Extensive genetic and genomics studies identified several major genes and QDRL for *P. sojae* resistance. The *Rps-*linked markers can be utilized in the selection of genotypes having PRSR resistance genes in early stages, and subsequent backcrossing will enable the rapid development of PRSR-resistant soybean cultivars. Marker-assisted breeding approaches such as MAGP can help in pyramiding vertical and horizontal resistance by the utilization of major resistance genes and QDRL identified in different genetic backgrounds. This strategy of combining complete and partial resistance in the same cultivars will prove to be the most effective approach in the near future. Soybean breeders need to continuously identify novel and unique resistance genes to cope with the emerging new pathotypes (Sugimoto et al., 2011). Though it is challenging to incorporate a large number of genes and QDRL from multiple genetic backgrounds into a single background using MABB, MARS and genomic selection can be used in resistance breeding programs to incorporate all PRSR resistance loci for durable resistance. It will be useful to mine the germplasm and geographical regions with enormous diversity for the presence of resistance to prevailing *P. sojae* pathotypes. For example, soybean germplasm collections in the Republic of Korea have greater variability for resistance to *P. sojae* for specific *Rps* loci, as well as partial resistance (Dorrance and Schmitthenner, 2000), and can be used for incorporating durable resistance through large-scale breeding programs. Emerging approaches such as gene discovery through re-sequencing, proteomics, metabolomics, RNA-seq, and exome sequencing of soybean and its wild relatives need to be exploited at a broader level. Furthermore, the QTL-seq approach will likely augment the rapid identification of novel QDRL and advancement of selected progenies for cultivar improvement (Zhang et al., 2018). The CRISPR/Cas9-mediated identification of effector-triggered immunity and *R*-gene editing is a highly targeted approach for the understanding and rapid development of PRSR resistance. Thus, different “Omics” approaches may be employed to explore the plant defense mechanisms in plant–pathogen interactions along with a gene-editing approach. In addition to the genetic improvement of cultivars for PRSR resistance, other alternative approaches need to be adopted and integrated to achieve prolonged resistance. Such approaches include the identification of effective compounds such as calcium that could help control the PRSR to certain levels (Sugimoto et al., 2010). Since the roots are primary targets for PRSR infection, the extensive comparative study of root traits in wild relatives or resistant cultivars to those of susceptible cultivars will help in the identification of certain target traits for phenotyping and resistance management. For such studies, phenotyping platforms that help in better visualization of root system architecture should be given high priority. The combined approach of genetic resistance, integrated disease management, and climate-smart agronomic practices can pave the path for the sustainable management of PRSR in soybean.
